# Construction and Validation of an Editable Image Dataset for Black Hole Effect Scenes in YOLO Object Detection

**DOI:** 10.3390/s26206615

**Published:** 2026-10-19

**Authors:** Jinyu Yang, Yun Chen, Shanhe Lou, Xinliang Geng, Zhongxiang Yang, Tengfei Wang, Yumei Liu, Xinyang Liu, Siying Li, Jinhe Lv, Qiushi Yan, Tie Li, Zhipeng Wang, Longchen Gong, Weirong Shu

**Affiliations:** 1 School of Civil Engineering and Transportation, Beihua University, Jilin 132013, China; yangjinyuchina@163.com (J.Y.); yiran_song2046@163.com (S.L.); 13179182725@163.com (X.G.); 17862270295@163.com (Z.Y.); wtf7826@163.com (T.W.); 18843715828@163.com (X.L.); 18649118323@163.com (S.L.); lvjinhe_1987@163.com (J.L.); yanqiushi2014@163.com (Q.Y.); litie0622@163.com (T.L.); a2429238362@163.com (Z.W.); 15981196980@163.com (L.G.); 15770307520@163.com (W.S.); 2 College of Transportation, Jilin University, Changchun 130025, China; lymlls@163.com

**Keywords:** computer vision, black hole dataset, autonomous driving, transfer learning, YOLO object detection

## Abstract

**Highlights:**

**What are the main findings?**

- A dedicated YOLO dataset is constructed for black hole effect scenarios at bridge and tunnel entrances, and an editable dataset management paradigm with semantic labels is proposed for flexible reuse.
- A 3D dynamic evolution model of black hole effect intensity is established using time–distance data.

**What are the implications of the main findings?**

- Standardized workflow and quality audit specifications for YOLO traffic dataset are established.
- Fine-tuning with the dataset improves YOLO mAP@0.5:0.95 by over 14.54% in black hole conditions.

**Abstract:**

The YOLO object detection algorithm is widely applied in intelligent assisted driving systems. However, existing datasets lack targeted coverage of the black hole effect scenarios at bridge and tunnel entrances. To address this issue, this paper constructs a black hole dataset, which consists of two subsets, namely BH-Normal and BH-Blackhole, involving four typical scenarios, namely suburban beltway tunnels, urban underground tunnels, railway overpasses, and interchanges, as well as multiple influencing factors such as different illumination angles and different distances from tunnel entrances. The dataset contains 27,868 labeled images in YOLO format, with 71,956 labeled instances. Based on statistical analysis of full-day time-series and distance-sequence data, this paper reveals the evolution pattern of black hole effect intensity at tunnel entrances with respect to observation distance and time of day. Meanwhile, this paper constructs an editable black hole dataset and develops a one-stop YOLO dataset creation and management toolkit. Baseline validation using mainstream full-scale YOLO models shows that the model fine-tuned solely on BH-Normal suffers an average mAP@0.5:0.95 drop of over 20% under black hole effect conditions; with the introduction of BH-Blackhole and secondary fine-tuning, the model’s mAP@0.5:0.95 is relatively improved by more than 14.54%. This confirms that the black hole dataset can effectively enhance the target detection performance of YOLO models in black hole effect scenarios at bridge and tunnel entrances. Furthermore, the baseline results of the YOLO model obtained in the black hole dataset can provide benchmark support for optimizing intelligent assisted driving visual perception models.

## 1. Introduction

**Figure 1:**
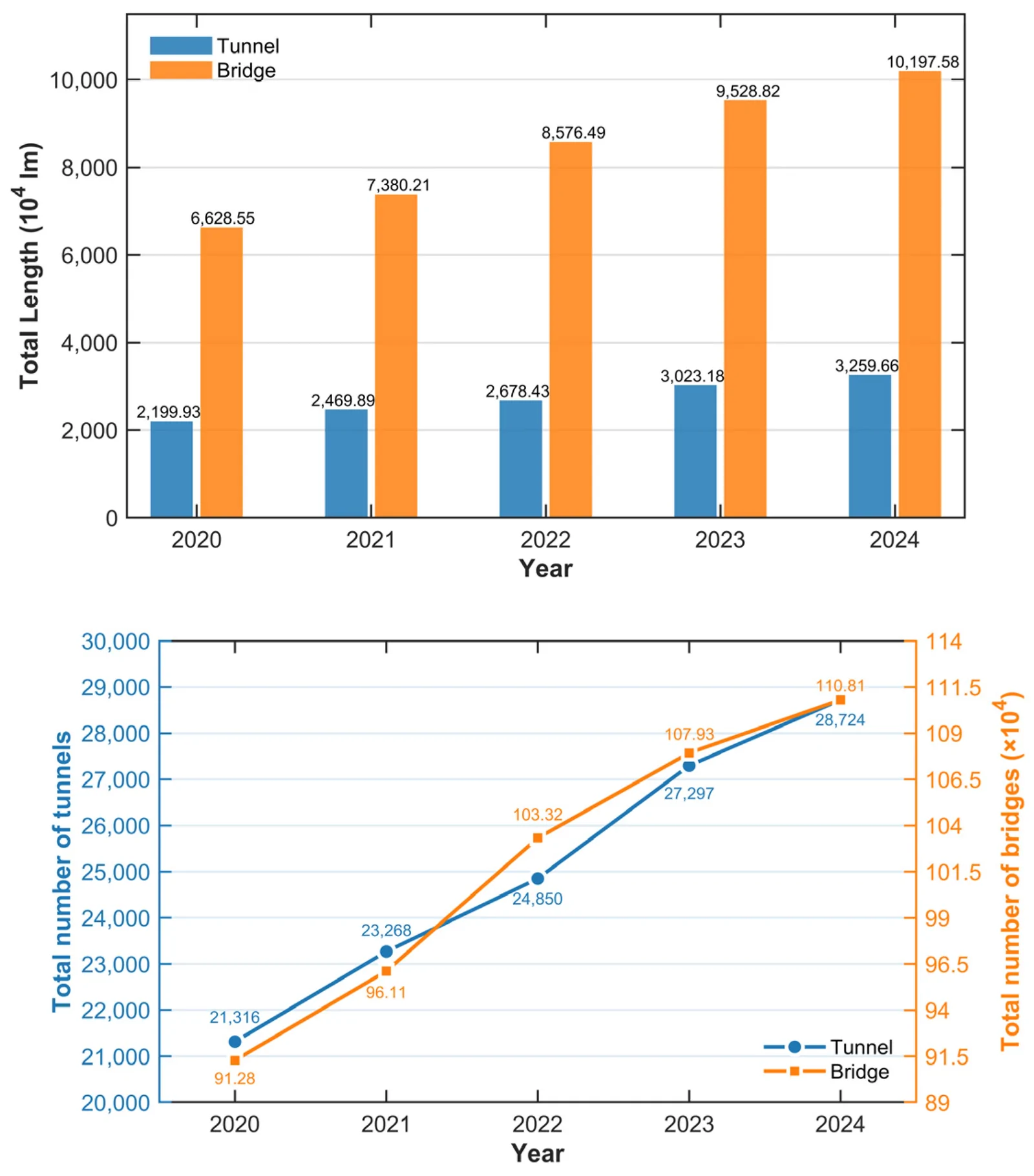
Mileage and quantity of tunnels and bridges in China (**upper**) and (**lower**).

With the continuous improvement of infrastructure construction level and the accelerated urbanization process, road infrastructure has become increasingly complex, leading to a sharp surge in the number of road scenarios susceptible to the “black hole effect”, such as tunnels, overpasses, and underpass interchanges. According to statistics, China added 195.3 thousand road bridges and 7408 road tunnels in the five-year period from 2020 to 2024, with a cumulative total of 1.1081 million road bridges and 28,724 road tunnels by the end of 2024 [1,2,3,4,5]. The specific changes in the number and mileage of bridges and tunnels are shown in Figure 1. The “black hole effect”, caused by drastic changes in ambient illumination at the entrances and exits of these scenarios, poses a significant challenge to both drivers and assisted driving systems in perceiving road conditions. To address this issue, existing solutions mainly include passive measures such as optimizing lighting and installing sunshades. However, these measures have drawbacks including high costs, poor effectiveness, and difficulty in later maintenance, and are difficult to cover rural roads and low-traffic sections. Therefore, relying on high-level assisted driving systems to autonomously overcome the black hole effect holds significant practical importance for enhancing driving safety, and it is also a crucial issue that urgently needs to be addressed.

In the field of advanced driver assistance systems (ADAS), two major technical routes have been formed: “pure vision” and “multi-sensor fusion”. Regardless of which route is adopted, camera-based object detection remains an indispensable core link. The YOLO series algorithms, with their excellent balance between accuracy and speed, not only guarantee recognition accuracy but also well satisfy the real-time requirements of assisted driving, thus becoming a focus of attention in both industry and academia. However, most existing studies are carried out based on general datasets such as MS COCO [6], which results in great challenges for object detection models under the condition of drastic illumination changes induced by the “black hole effect”. Specialized datasets for traffic scenarios, such as BDD100K [7], also fail to meet the training requirements due to the scarcity of samples in low-illumination bridge and tunnel environments. Therefore, constructing a dataset that helps YOLO object detection algorithms tackle the “black hole effect” and improve the detection accuracy of assisted driving models has become an urgent task.

Existing research on the black hole effect is still largely limited to static descriptive analyses. From the perspective of engineering standards, the International Commission on Illumination CIE 88:2004 standard [8], although serving as the authoritative basis for tunnel lighting design, calculates the brightness parameter L_20_ of the entrance section based solely on a single position corresponding to the Stopping Sight Distance (SSD), thus embodying a static design paradigm. From the perspective of traffic behavior research, existing studies have collected multi-distance illuminance data inside and outside tunnels and analyzed drivers’ pupil size changes and mental workload, but these studies are still limited to cross-sectional measurements at specific time points, failing to effectively reveal the temporal influence mechanism of dynamic solar altitude angle changes on the black hole effect. In recent years, a few studies have attempted to integrate both time and distance factors into investigations related to the black hole effect. However, all of them have significant limitations, making it difficult to achieve refined and quantitative characterization of the evolution law of the intensity of the black hole effect.

Most existing datasets are released in an “integrated” packaging format. While this offers certain convenience for dataset utilization, its monolithic and rigid architecture restricts researchers from flexibly invoking data in sub-scenarios, subcategories, or subtasks, thereby posing significant obstacles to personalized research with fragmented requirements. Even though attempts have been made to split some datasets, their subset labels still lack semantic information and cannot provide targeted data filtering guidance for subsequent tasks. COCO is a widely recognized performance evaluation benchmark for current object detection tasks. It contains approximately 330,000 annotated images, covering a total of 80 object categories. However, traffic-related objects (e.g., cars, bicycles, buses, and trucks) account for merely less than 8% of the total instances. Moreover, the official annotations do not include labels such as “tunnel” or “overpass”, nor does it provide any sampling strategy tailored to the black hole effect. Therefore, general datasets cannot cover the extreme low-light and high-contrast scenarios unique to tunnel entrances, thus failing to support the robustness evaluation of YOLO series algorithms in such special scenarios. Datasets in the traffic domain also lack specificity for this task. KITTI [9] explicitly states in the original paper that it has excluded scenarios with poor lighting conditions (e.g., tunnels). Although Cityscapes [10] classifies “tunnel” as a semantic category under the “construction” group, it does not disclose the specific proportion of such scenarios, which can only be inferred from the provided pixel counts under fine-grained annotations to be extremely low. While BDD100K contains 100K annotated high-resolution frames with scene labels, only 206 segments are labeled with “tunnel”, accounting for merely 0.206% of the total segments. In summary, existing traffic datasets are basically non-existent in the subfield focusing on the impact of the black hole effect. Existing image data of tunnel segments can be roughly categorized into two types: long-term recordings of the tunnel interior captured by fixed surveillance cameras [11], which are used for traffic flow statistics or event detection, and short-term recordings captured by vehicle-mounted front-facing cameras, which still focus on vehicles and pedestrians inside the tunnel. Both types of data are collected inside the tunnel body, where the illumination conditions are relatively stable, and do not involve the drastic changes in illumination intensity between the inside and outside of the tunnel entrance.

Up to now, there is no public dataset that includes a dedicated subset for the extreme scenarios at tunnel entrances, a problem that severely hinders the accuracy improvement of YOLO-based object detection in such extreme environments. Therefore, BH constructed in this paper systematically covers the bridge and tunnel entrance scenarios for the first time, providing a reliable benchmark for model training and evaluation, thereby effectively filling the data gap in this field.

The research on the “black hole effect” in road scenarios primarily focuses on tunnel entrance regions, and its quantitative evaluation and data support can be categorized into two core dimensions: engineering technical design and traffic behavioral response. From the perspective of engineering technology, the standard CIE 88:2004 formulated by the International Commission on Illumination (CIE) provides a fundamental guideline for tunnel lighting design worldwide. Subsequently, CIE 193:2010 [12] further supplements the specifications of emergency lighting configuration, installation, operation, and maintenance, forming a universally accepted framework for tunnel lighting design. Nevertheless, in these specifications, the key parameter *L*_20_ for tunnel entrance luminance calculation is only required to be measured at a single location corresponding to the stopping sight distance, with the measurement duration limited to 75 cumulative daylight hours with the highest ambient brightness throughout the year. Although the specification mentions the principles of dynamic sunlight variation and adaptive lighting control, it fails to provide quantitative data to support its practical feasibility. Jinghang Xiao et al. [13] adopted the measurement method specified in the standards and revealed the temporal varying characteristics of *L*_20_ in east–west-oriented tunnels via field tests, i.e., the luminance peak appears in the morning for eastbound tunnels and in the afternoon for westbound tunnels. However, this study still fails to break through the limitation of single-point measurement and cannot characterize the luminance gradient variation at different distances from the tunnel entrance.

In terms of traffic behavior, existing studies predominantly analyze the influence of the black hole effect based on drivers’ physiological and behavioral responses. Hanlin Xu et al. [14] conducted virtual reality experiments and quantified the impact of the black hole effect on drivers’ psychological stress and driving performance at different distances from tunnel entrances using indicators such as pupil diameter variation. Mingxiu Liu et al. [15] established the correlation between illuminance inside and outside tunnel entrances and drivers’ pupil area through on-road vehicle tests, verifying the association between sudden illuminance transitions and visual discomfort. Nevertheless, both studies are confined to the single spatial variable of distance while ignoring temporal dynamic variations (e.g., daylight intensity differences across different time periods). Yangjian Yu et al. [16] explored the spatiotemporal variation in ambient correlated color temperature (CCT) within drivers’ 20° field of view through field dynamic tests and identified the 50 m position at tunnel entrances as the CCT peak point. In fact, the black hole effect is dominated by multifactorial coupling effects; single CCT is not recognized as a mainstream evaluation indicator and cannot fully characterize its comprehensive impact on driving safety. Bo Liang et al. [17] acquired data on drivers’ average reaction time at different distances from tunnel entrances and saccade speed in different time periods via static simulation and on-road vehicle tests, preliminarily revealing the spatiotemporal variation patterns of the effect. However, their work has obvious spatiotemporal scale defects: distance is measured in kilometers and the time interval is set to 2 h. Such overly coarse measurement scales cannot capture the drastic fluctuations of the black hole effect over short distances (tens of meters) and short time scales (minute-scale) at tunnel entrances, and thus cannot accurately reflect its influence on drivers’ instant driving decision-making. Addressing the deficiencies and gaps in existing research, this paper systematically collects and constructs a fine-grained spatiotemporal sequence data for suburban beltway tunnels. Based on this dedicated data, a three-dimensional dynamic evolution model for characterizing black hole effect intensity is preliminarily established from temporal–spatial dual dimensions. The findings offer precise quantitative support for the subsequent acquisition, preprocessing, and other downstream tasks of BH.

In the management paradigms of datasets, existing public datasets commonly adopt an all-in-one encapsulation strategy. Taking COCO 2017 as an example, all samples are released in unified packages categorized into train, val, and test subsets. While this approach reduces the usage threshold, its large volume leads to coarse data granularity, making it difficult to support fine-grained subset filtering and targeted analysis. Autonomous driving scenario datasets involve multiple types of sensors (cameras, light detection and ranging, etc.), and thus are usually partitioned into multiple sub-datasets based on acquisition type or research task. ONCE [18] further subdivides the train, validation, and test splits into two subcategories: camera and light detection and ranging data. ApolloScape [19] is divided into independent subsets such as Scene Parsing, 3D Car Instance Understanding, and Lane Segmentation according to research tasks. On this basis, ApolloScape further clarifies the folder structure of the public dataset, subdividing it according to labels such as data type, road ID, and timestamp. Although this “labeling + modularization” approach significantly improves retrieval and invocation efficiency, the existing labels still lack sufficient coupling with the data content. Taking ApolloScape as an example, although it covers multi-weather scenarios (e.g., rainy, snowy, and sunny conditions), the key attribute “weather” is not annotated or recorded during data management. Researchers requiring only the rainy-day subset must invest considerable time and effort. E-BH inherits and advances the aforementioned “labeling + modularization” paradigm, and further enhances the deep integration of labeling and data content diversity. It allows researchers to quickly extract samples with gradient intensities of the “black hole effect” using timestamps, or recombine subsets based on labels (e.g., scenario type and target distance), thereby facilitating controlled experiments focused on the model’s scale generalization capability. This mechanism fully taps into the research value of data diversity, laying a solid foundation for subsequent secondary development and personalized combination.

This paper first collected full-day time-series and continuous distance gradient image data at the entrance of the suburban beltway tunnel. Through statistical analysis of the collected data, a three-dimensional dynamic evolution model of the black hole effect intensity under the time space dual dimension was established, which provides a theoretical basis for data collection and dataset subset division in other bridge tunnel scenarios. Meanwhile, this paper summarizes and establishes a complete YOLO dataset construction process and quality audit specification, clarifying the problems of ambiguous processes and lack of standards in traditional dataset production. This specification is of great guiding significance for the standardized construction of high-precision datasets, especially those for traffic scenarios. Subsequently, based on the aforementioned research, this paper completed the construction of BH (the Black Hole dataset). This dataset contains over 27,000 finely labeled images, covering four typical scenarios with a high incidence of the black hole effect: suburban ring road tunnels, urban underground tunnels, railway overpasses, and interchanges. It is divided into BH-Normal and BH-Blackhole subsets according to whether the detected targets are affected by the black hole effect. Addressing the issues of rigidity and poor reusability inherent in the aforementioned monolithic data, this paper proposes a modular management model that closely integrates data labels with data content and characteristics. Based on this, E-BH (the Editable-BH dataset) has been created.

Additionally, TK (a one-stop YOLO dataset creation and management toolkit) has been developed, integrating a comprehensive set of practical scripts covering data processing, label review, and secondary development. This provides strong support for the community to efficiently reuse, continuously iterate, and expand the BH. Finally, this paper conducted dataset baseline comparison experiments on the constructed BH using full-scale models of mainstream YOLO architectures (v5, v8, 11, 12, 26) with all scales (n, s, m, l, x) [20,21]. In the experiments, we first fine-tuned each YOLO model based on the BH-Normal subset to construct the BH-N model set and then performed secondary fine-tuning on the BH-N model set using the BH-Blackhole subset to obtain the BH-B model set. By comparing the test performance of the two model sets under black hole effect scenarios, it is effectively verified that BH constructed in this study can significantly improve the object detection performance of YOLO models under the interference of the black hole effect.

Our contributions are summarized as follows:

- Based on field measured data, this paper constructs a three-dimensional dynamic evolution model for the intensity of the black hole effect at tunnel portals from both temporal and spatial dimensions. For the first time, from the perspective of onboard images of autonomous vehicles, this work reveals the dynamic evolution mechanism of black hole effect intensity under the coupled action of temporal and distance factors. The findings can not only provide theoretical support for the acquisition specification and category classification of datasets in typical bridge-tunnel traffic scenarios, but also serve as a reference for the planning, construction and portal structural optimization design of bridge-tunnel engineering.
- Aiming at typical bridge-tunnel entrance scenarios, this paper establishes a real-scene YOLO object detection dataset named BH, which effectively bridges the gap in available dedicated datasets for the research on the black hole effect at tunnel portals among existing public databases. In the dataset construction procedure, a standardized workflow and quality audit specification for YOLO traffic dataset development are formulated, which remedies the problems of non-standard production procedures and imperfect quality control systems of current traffic datasets. Meanwhile, a management paradigm integrating modular dataset architecture and semantic subset labels is proposed. The development of E-BH and TK provides a feasible paradigm and technical support for the standardized management and lightweight secondary development of YOLO datasets.
- By conducting baseline evaluations of mainstream full-scale YOLO architectures on BH, this paper not only validates the effectiveness of BH in boosting the object detection accuracy of YOLO models under bridge-tunnel black hole effect scenarios but also provides a reliable data benchmark and experimental baseline for subsequent algorithm optimization and model improvement of YOLO in such scenarios.

The remainder of this paper is structured as follows. Section 2 elaborates on the procedures and results of dataset collection, preprocessing, annotation and statistical analysis. Section 3 introduces baseline experiments of mainstream full-scale YOLO architecture models on BH, and analyzes the experimental results. Finally, Section 4 summarizes the entire work.

## 2. Methods

**Figure 2:**
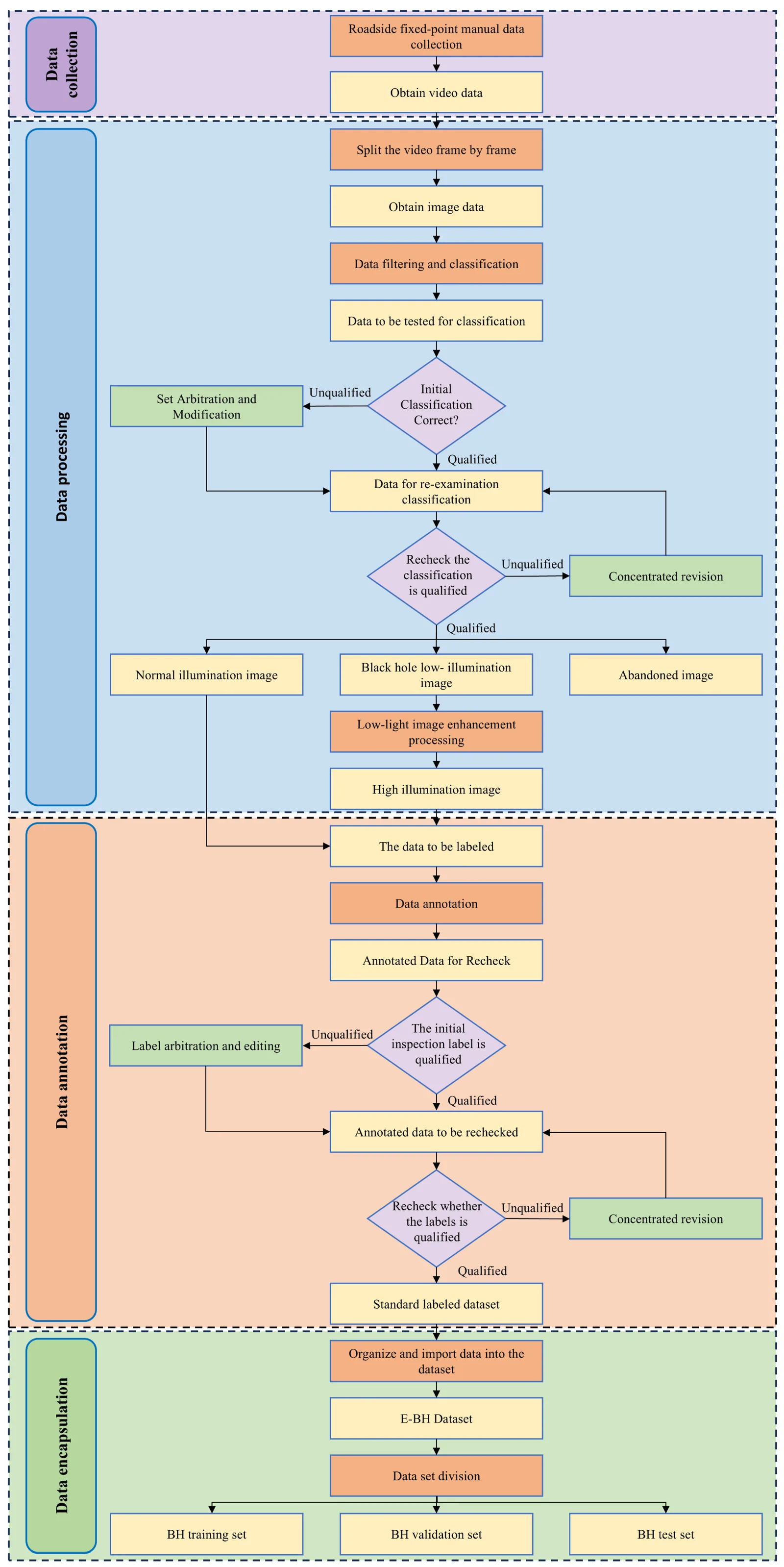
Data set creation flowchart.

Data collection in this study was performed in winter, with collection location selected including suburban beltway tunnels in Zhangjiakou City, Hebei Province, China, and three typical traffic scenarios in urban Jilin City, Jilin Province, China: urban underground tunnels, railway overpasses, and interchanges. The dataset construction process was as follows: First, raw video data was obtained through manual fixed-point roadside collection. Then, data processing was performed, which involved video frame extraction and conversion into images, image selection and classification, and low-light image enhancement. Subsequently, fine-grained annotation was conducted on the image samples. Finally, dataset encapsulation and statistical analysis were completed, and the overall technical workflow is shown in Figure 2. In addition, this study further developed E-BH, a modular version of the BH, and integrated a dedicated supporting toolkit, which provides technical support for the secondary development of the BH.

### 2.1. Data Collection

The speed limits on all data collection sections do not exceed 40 km/h. According to China’s “Technical Conditions for Motor Vehicle Operation Safety”, the required braking distance for fully loaded passenger cars at an initial braking speed of 50 km/h is no more than 20 m [22]. As calculated by Equation (1), the theoretical full braking distance of vehicles traveling at the maximum speed limit of the sections is 12.8 m. In the formula, v denotes the final velocity, a represents the acceleration, and x is the displacement. Previous studies have indicated [23] that drivers’ emergency braking response time follows a normal distribution with a mean of 1.96 s. During this stage, the vehicle maintains its original speed, covering a distance of 21.78 m. In addition to the above, the braking process also includes the time it takes for the brakes to take effect. Meanwhile, braking distance is also affected by factors such as road adhesion and tire wear. Taking into account these influencing factors, a 15% redundant safety design is implemented. This paper finally defines the data collection range as the area within 40 m before the tunnel entrance.

*v*^2^ = 2*ax* (1)

**Figure 3:**
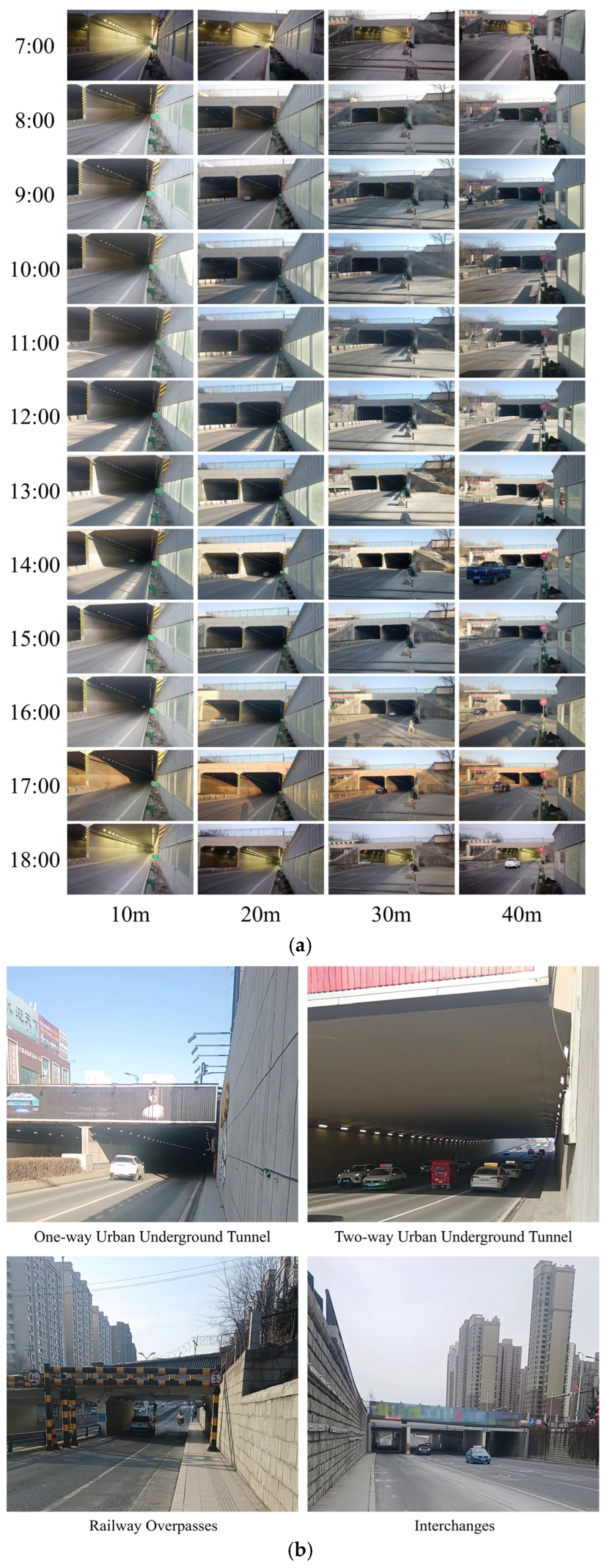
Collected data. (**a**) Time–distance image data. (**b**) Four scenarios for collecting data images.

The BH first selects suburban tunnel entrances with the most prominent black hole effect as the data collection scenarios. The initial sampling interval is set to 30 min, with a single collection duration of 10 s, covering the tunnel entrance traffic operation conditions under different natural lighting conditions throughout the 7:00–18:00 time period. Collection points are arranged in the range of 10–40 m outside the tunnel entrance, with sampling points spaced at 10 m intervals, which fully reproduces the real driving perspectives and driving states of vehicles gradually entering the tunnel entrance at different time periods. Partial data images are presented in Figure 3a. Statistical analysis of the complete time–distance sequence sampling data was conducted, which provides a scientific basis for the subsequent data collection scheme.

During the formal collection of the dataset, the single collection duration is adjusted to 5–6 min. The collected video data cover sample acquisition across multiple all-day illumination periods and distance dimensions. In addition, targeted data supplementation is conducted for the high-illumination periods and distance intervals where the black hole effect is prominent. While ensuring the overall generalization ability of the dataset, it effectively improves the sample proportion and scenario representativeness of typical tunnel black hole effect scenarios.

To further diversify scene categories and enhance the complexity and practical applicability of the BH, three typical urban road structure scenes susceptible to inducing the black hole effect are additionally collected: urban underground tunnels, railway overpasses, and interchanges, as illustrated in Figure 3b. This study ultimately collected 199 video segments in total, with a total duration exceeding 600 min, covering two recording resolutions: 1080 × 1920 (vertical) and 1920 × 1080 (horizontal).

### 2.2. Data Processing

(1) Video Frame Extraction to Image Data

The original video data is captured at a frame rate of 30 fps. To reduce computational overhead and preserve critical spatiotemporal information, this paper uniformly samples 3 frames per second to convert video sequences into image sequences. To avoid quality degradation induced by encoding and compression, all frames are stored in lossless PNG format, yielding a final dataset of 106,977 images.

(2) Data Filtering and Classification

First, images without annotation targets, images blurred due to the rapid movement of targets, and images containing only targets that are too far beyond the annotation range are designated as discarded images. The remaining images are classified based on whether the targets are located at the entrances of bridges and tunnels and thus affected by the black hole effect; they are further categorized into normal illumination images and black hole low-illumination images, respectively. Although railway overpass scenarios form local low-illumination areas due to bridge occlusion, the limited width of the bridge exerts no significant impact on target observation; therefore, all images in this scenario are designated as normal illumination images.

The classification process adopts a multi-level quality control mechanism. First, data classifiers perform preliminary processing on the images to generate pending classification data. Next, classification verifiers conduct preliminary inspections. Data that passes the initial inspection is marked as data pending recheck. For data failing the initial inspection, joint evaluation and correction by data classifiers and classification verifiers are required before it is also marked as data pending recheck. All pending recheck data is jointly reviewed and verified by classification verifiers and the chief labeling verifier to ensure that the screening results conform to labeling specifications. Images that fail the final review are centrally revised and re-entered into the recheck workflow, and this process is iterated until all images pass inspection. Finally, all qualified images are divided into three categories: normal illumination images, black-hole low-illumination images, and discarded images.

(3) Low-Illumination Image Enhancement

Black hole low-illumination images are subject to the black hole effect, characterized by blurred target details and extremely low contrast, posing significant challenges to subsequent annotation work. In response to this issue, this paper adopts the Multi-Scale Retinex with Color Restoration (MSRCR) algorithm [24] to perform batch processing on the images, thereby effectively improving image illumination, enhancing detail contrast, and providing clear and reliable image data support for subsequent annotation tasks.

MSRCR is a classic low-illumination image enhancement method based on Retinex theory. Retinex theory assumes that the image *S*(*x*,*y*) perceived by the human eye is the product of the illumination component *L*(*x*,*y*) and the reflection component *R*(*x*,*y*), as shown in Equation (2). Retinex theory transforms the product relationship into a difference relationship through logarithmic domain transformation, thereby separating and suppressing the illumination component and restoring the true reflection information of the scene.

*S*(*x*,*y*) = *L*(*x*,*y*) ⋅ *R*(*x*,*y*) (2)

**Figure 4:**
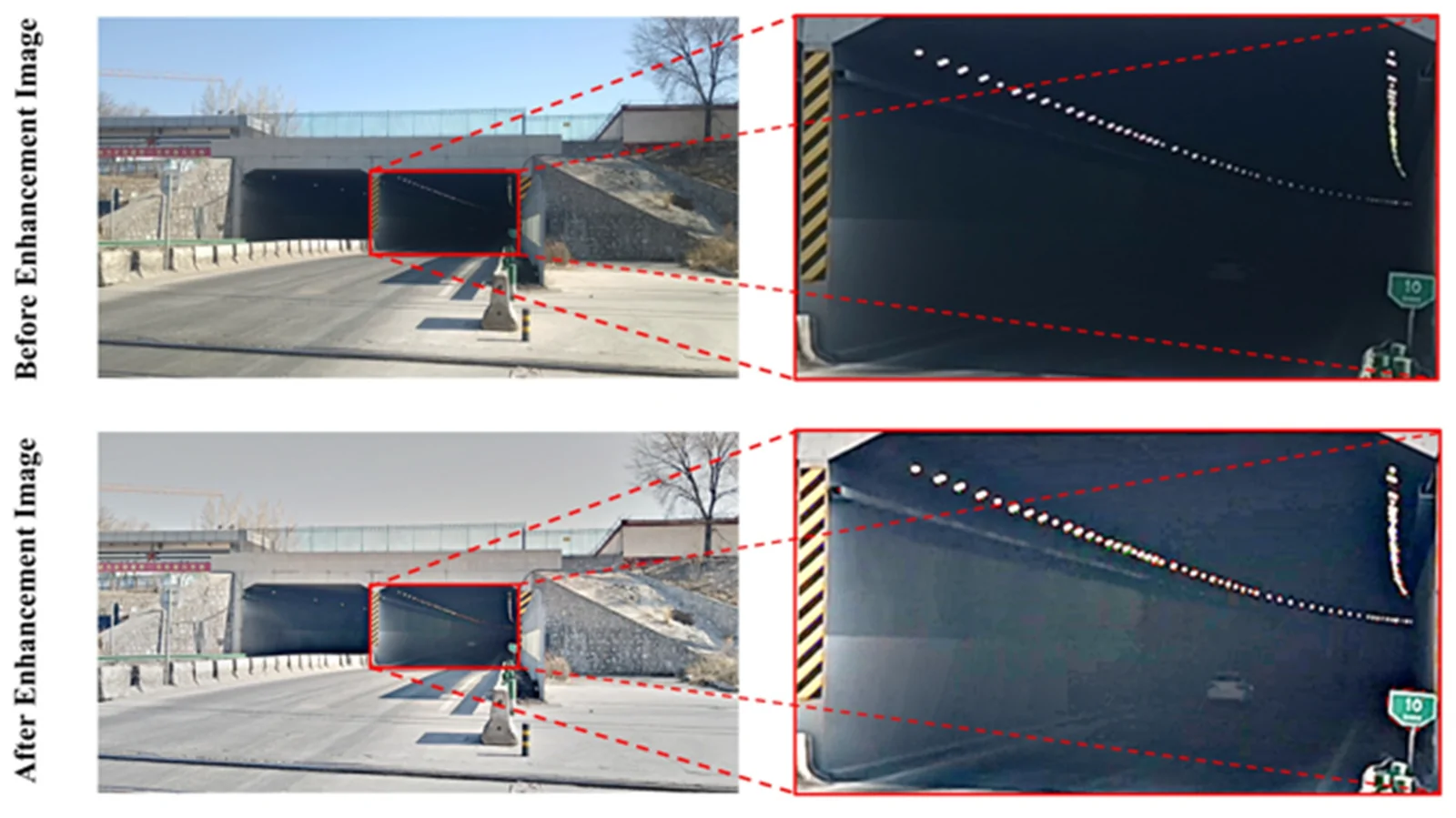
Comparison of low-light image enhancement before and after.

MSRCR introduces multi-scale Gaussian filtering based on Single-Scale Retinex (SSR), achieving a balance between local details and global illumination consistency by fusing Retinex results at multiple scales. Furthermore, the method introduces a Color Restoration Function (CRF) to dynamically adjust the gain value according to the relative intensity of each color channel, effectively suppressing color distortion and color cast artifacts common in traditional Retinex methods. Finally, after gain adjustment and histogram truncation normalization, the results are mapped to the display range. The comparison of images before and after MSRCR enhancement is shown in Figure 4.

### 2.3. Data Label

**Figure 5:**
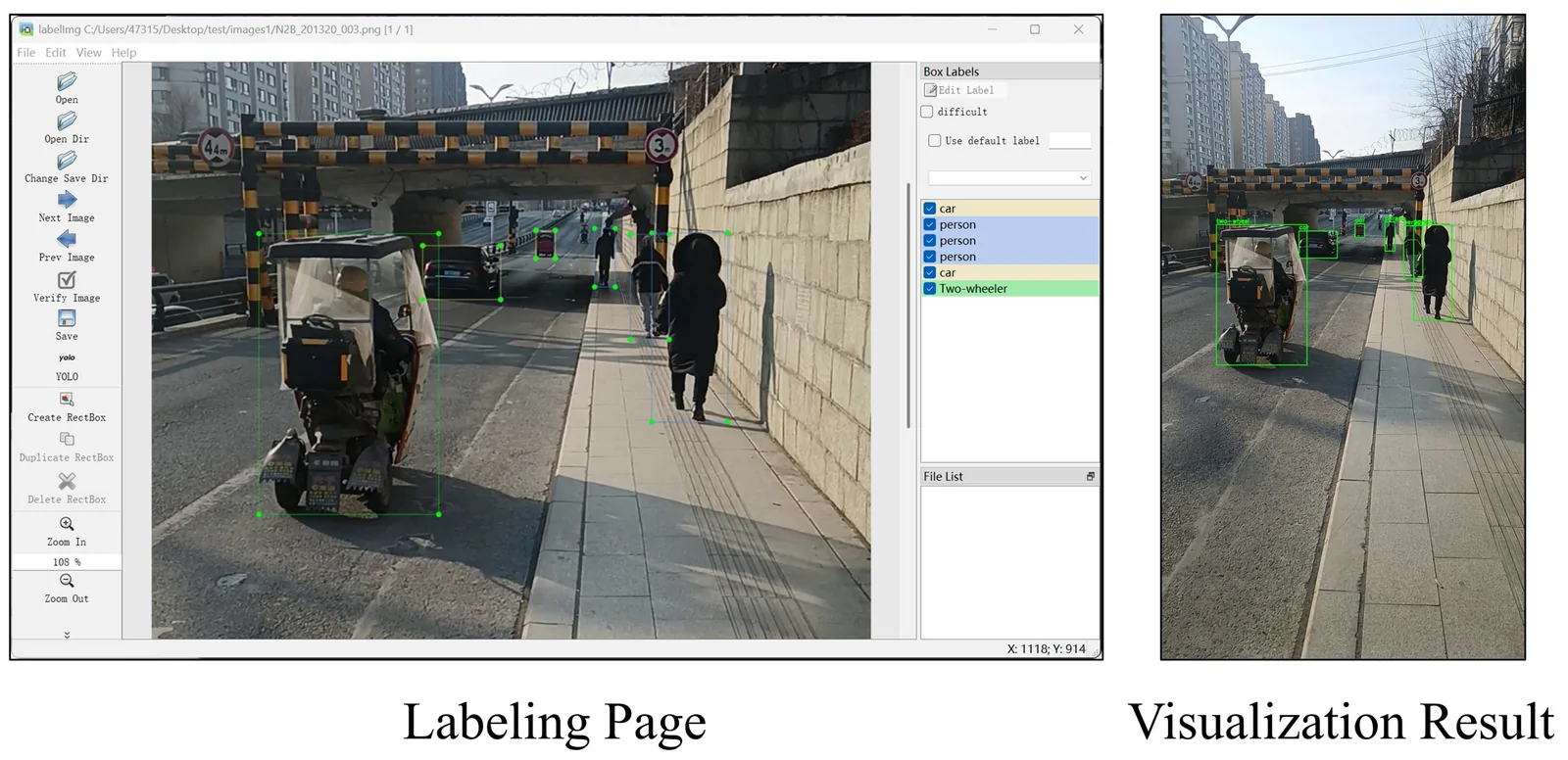
Label page and label visualization results.

Image enhancement samples and normal illumination images were uniformly incorporated into the data to be labeled. In this study, the LabelImg label tool was employed for target bounding box labeling, generating .txt format label files that can be directly adapted for YOLO algorithm training. The labeling results and their visualization are shown in Figure 5. Based on multiple characteristic dimensions, including vehicle type, size, and driving speed, the labeled targets were divided into four categories: car, truck, two-wheeler, and person. Specifically, electric vehicles, bicycles, and elderly mobility three-wheeled vehicles were clustered into the two-wheeler category. This classification method effectively mitigates the model instability caused by small-sample category data.

Before the formal labeling work is carried out, label consistency verification was performed on labelers and label verifiers to ensure their consistency in labeling. In this study, images were sampled from various scenarios and working conditions to be labeled, so as to construct a pre-labeling dataset consisting of 300 samples. A standard version of the pre-labeling dataset was jointly developed by third-party professionals. Subsequently, the built-in program of TK was used to quantitatively compare the differences between the labeling results of labelers and verifiers and the standard version. Through multiple rounds of iterative correction and unified standardization, the label deviation caused by human subjective biases was minimized, thereby ensuring the consistency and reliability of the labeling results.

**Figure 6:**
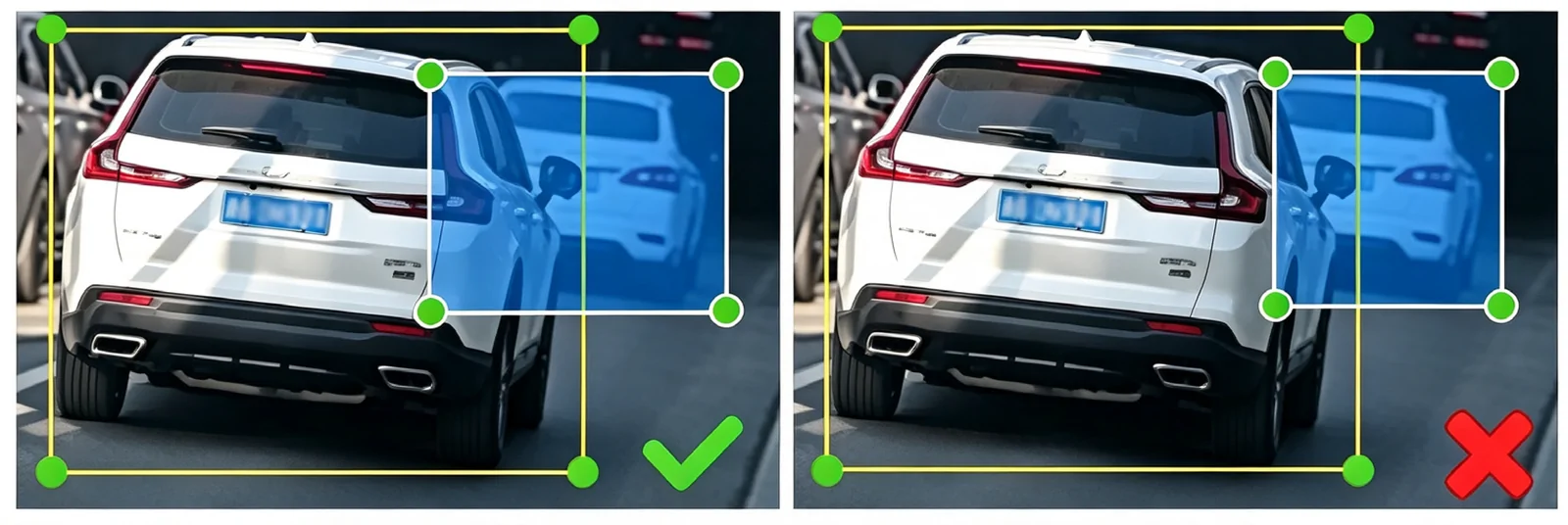
Standard labeling schematic.

During data labeling, the image data to be labeled was first labeled by professional labelers. During the labeling process, labelers were required to make the target bounding box closely adhere to the complete contour edge of the object. For occluded targets, the complete contour range was reasonably estimated based on the inherent shape of the target, and the labeling standard is shown in Figure 6. Based on long-term labeling experience, consistent constrained labeling of the same target was achieved by associating consecutive frames, which significantly improved the labeling accuracy of occluded targets and vehicle edge contours. After the initial labeling, the data was transferred to the verification stage as data to be verified. Subsequently, multiple label verifiers performed verification on the LabelImg platform: samples with compliant labeling were classified into the dataset to be rechecked; samples with issues such as missing labels, redundant labels (including redundant labels of invalid targets with blurred distant features), and excessive bounding box offset were deemed unqualified. These unqualified samples were jointly reviewed and arbitrated by labelers and verifiers, corrected, and then uniformly included in the dataset to be rechecked. Finally, the data to be rechecked underwent a standardized review by the chief label verifier using the built-in label visualization program of the TK, which effectively suppressed excessive label offset of the same target in consecutive images. Samples that failed the review were centrally rectified and re-entered the recheck process for cyclic verification. This iterative optimization was continued until all labeled samples passed the review, resulting in a high-quality usable dataset.

### 2.4. Data Packaging

In the data encapsulation stage, all the coordinates of the annotation boxes are mapped and saved onto the corresponding original images. The enhanced images used to assist annotation are then discarded and do not enter the final dataset. The original images and annotation files that have been verified as correctly matched will be organized into a dataset in a standardized format.

**Figure 7:**
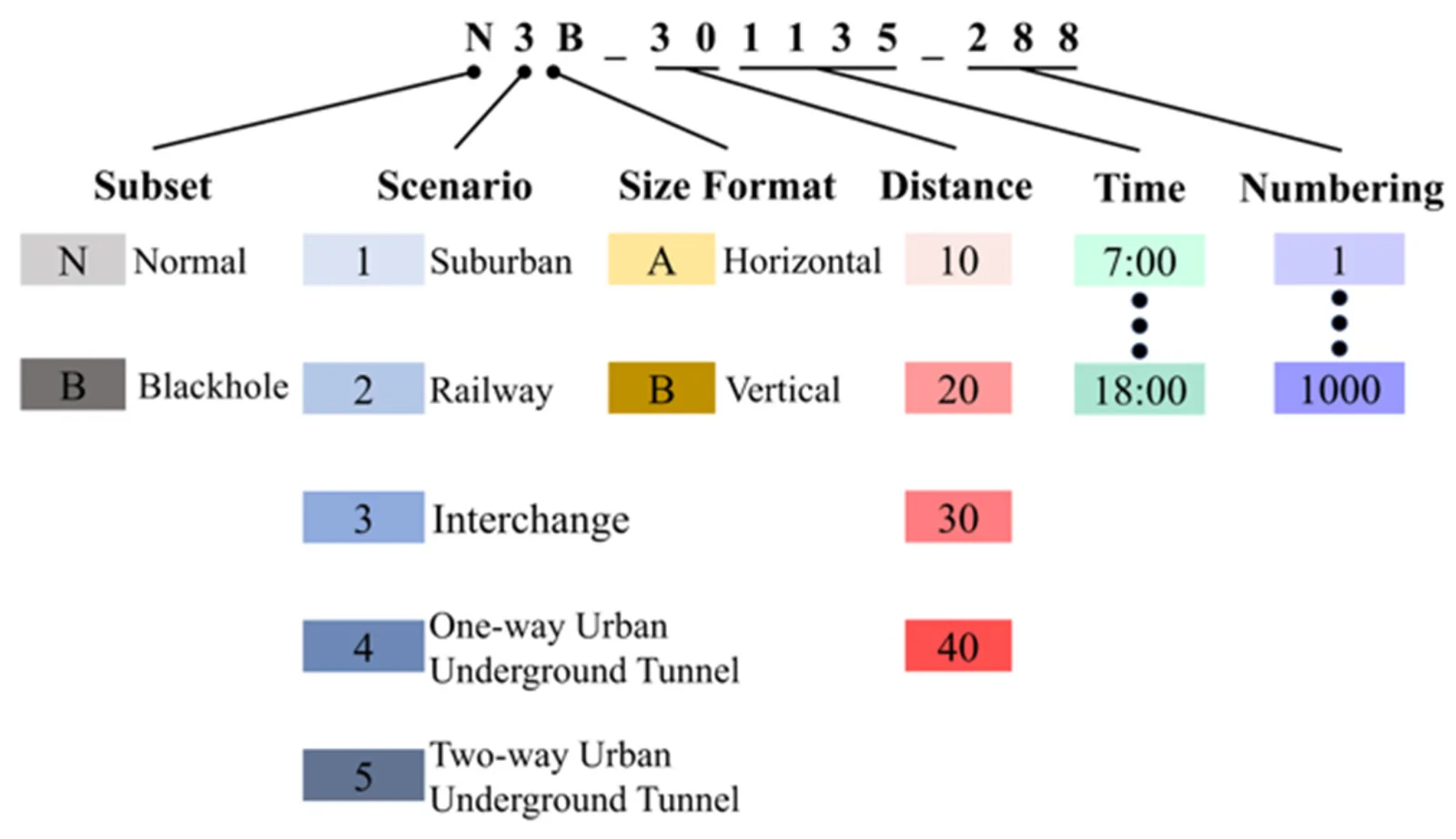
Naming method for image retrieval.

The dataset is managed through structured classification across multiple dimensions: it is divided into the Normal subset and the Blackhole subset (characterized by black hole effect interference) based on whether the images contain black hole effect interference targets; categorized into five types according to shooting scenarios, namely suburban beltway tunnels, railway overpasses, interchanges, one-way urban underground tunnels, and two-way urban underground tunnels; classified into horizontal and vertical orientations according to the shooting resolution (1920 × 1080 and 1080 × 1920); and further finely divided by the distance from the bridge-tunnel entrance (10 m, 20 m, 30 m, 40 m) and shooting time window (7:00–18:15). All images in the dataset are uniformly named in accordance with the aforementioned multi-dimensional attributes, with the naming example shown in Figure 7. This packaging and classification management approach can fully characterize the attributes of individual samples, clearly distinguish differences among various types of data, facilitate rapid filtering and retrieval by subsequent researchers, and provide solid support for the reuse, expansion, and secondary development of the dataset.

**Figure 8:**
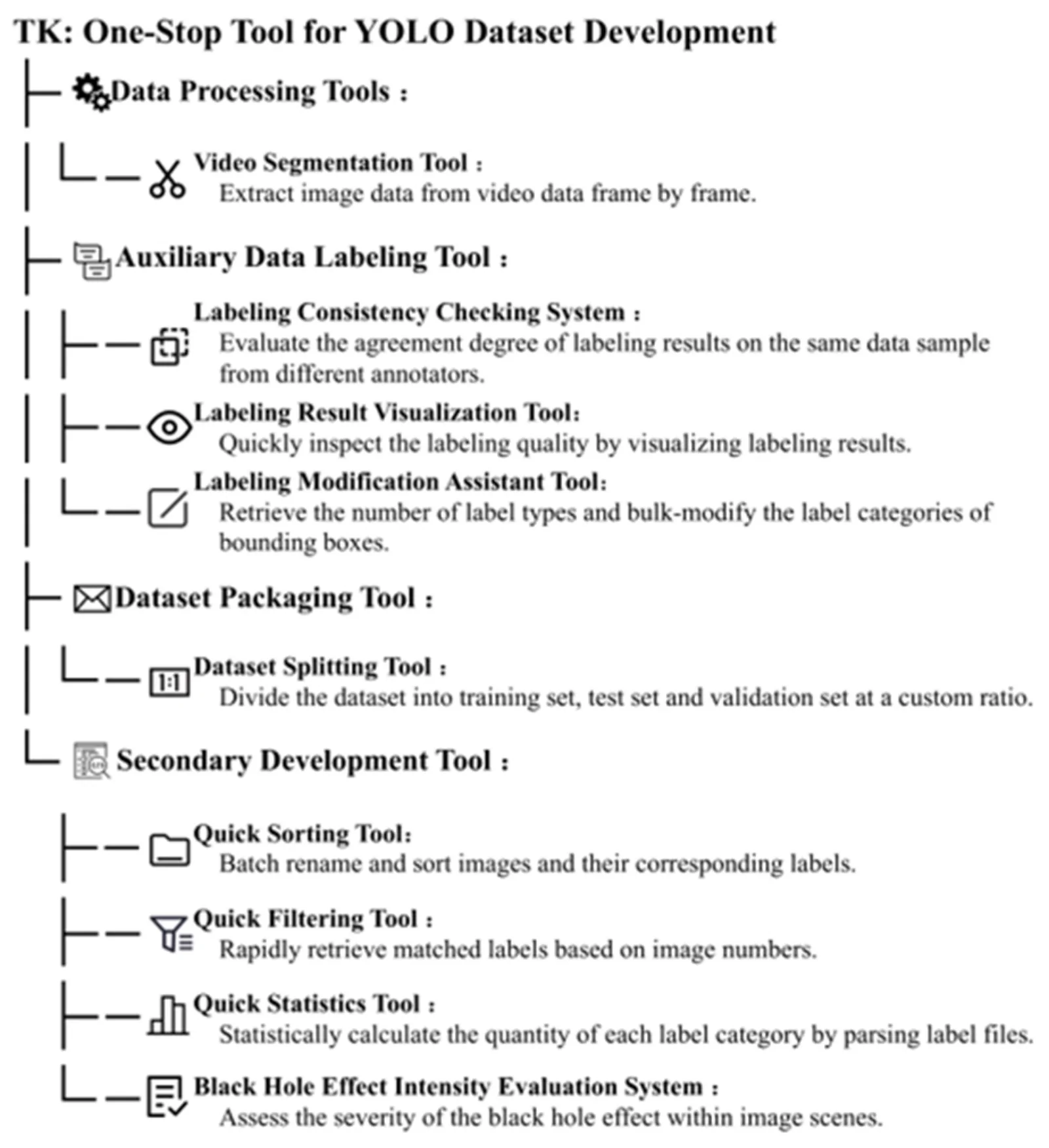
TK’s built-in program tree.

To facilitate subsequent research and development, this paper develops the TK, which covers a full-process program package encompassing data processing, data annotation assistance, data packaging, and secondary development, and provides one-stop development support. The specific functions and roles of each module are shown in Figure 8.

BH is constructed by merging the E-BH, comprising two subsets: BH-Normal and BH-Blackhole. Using the built-in program of the TK, images from both subsets were randomly split into training, validation and test sets at a ratio of 8:1:1.

### 2.5. Data Statistics and Analysis

**Figure 9:**
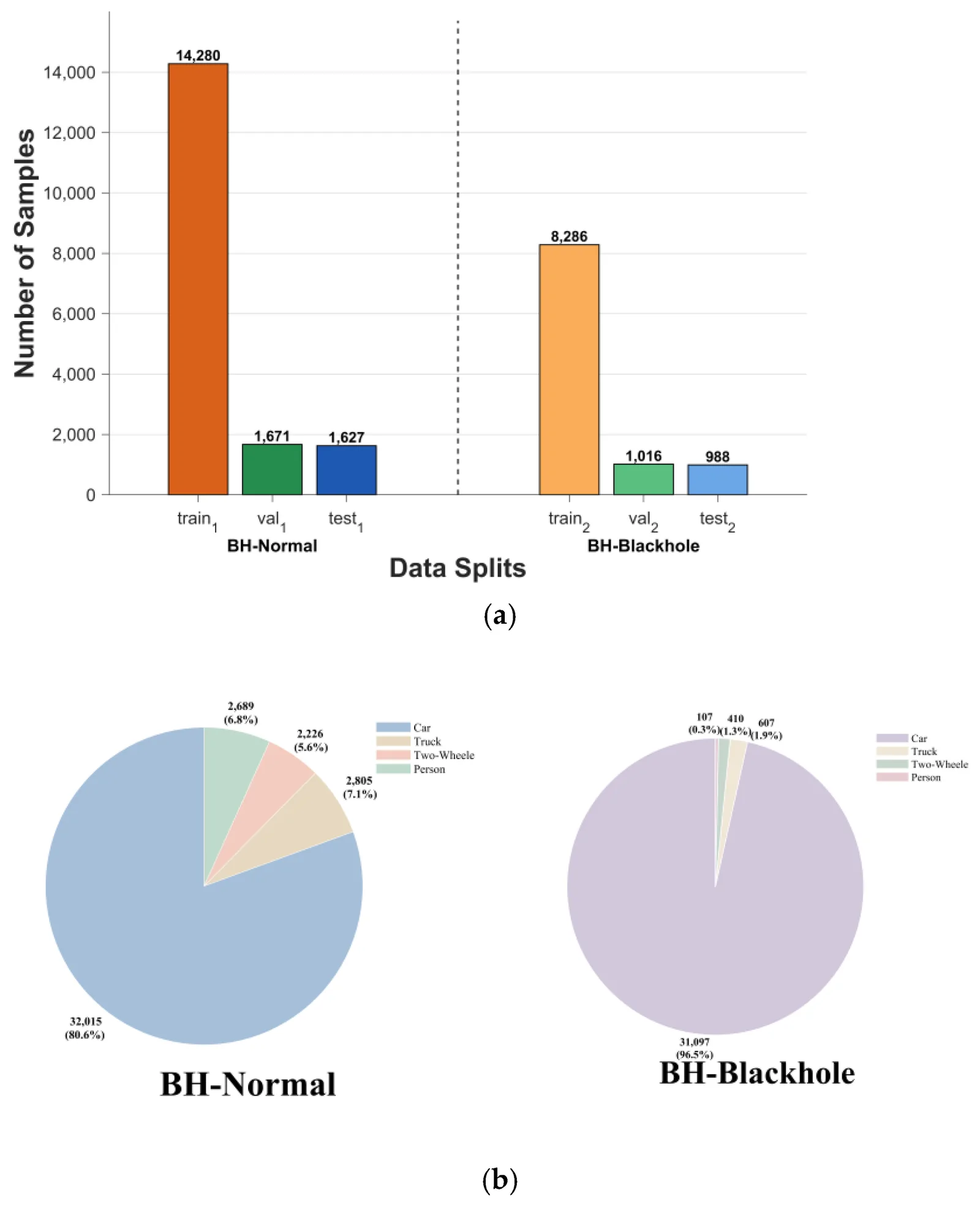
Data Statistics. (**a**) Statistics on the number of data segments. (**b**) Number and percentage of tag categories.

BH consists of 27,868 images with corresponding label files, which are categorized into two subsets, namely BH-Normal and BH-Blackhole, containing 17,578 and 10,290 samples, respectively. Both subsets are partitioned into training, validation, and test sets, and the detailed sample distribution of each subset is illustrated in Figure 9a. Label statistics on BH yield a total of 71,956 valid labels, covering four object categories: car, truck, two-wheeler, and person. The sample count and proportion of each category are 63,112 (87.71%), 3412 (4.74%), 2636 (3.66%), and 2796 (3.89%), respectively. The category distribution across different subsets is presented in Figure 9b.

**Figure 10:**
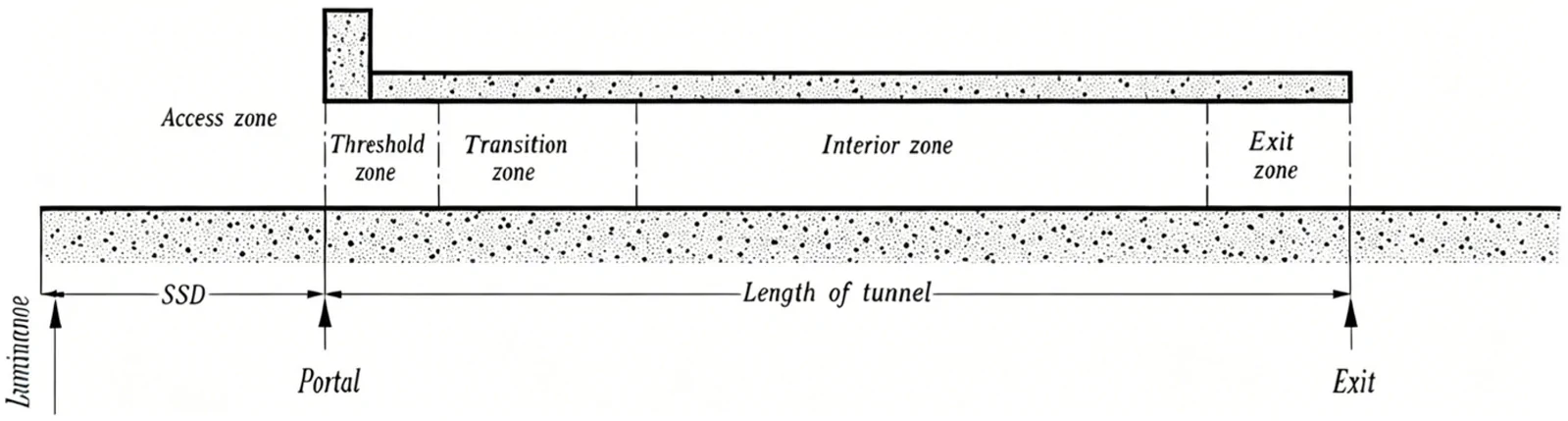
Zones in a tunnel.

In accordance with the CIE 88:2004 standard, a tunnel is divided sequentially into threshold segment, transition segment, interior segment and exit segment from the entrance to the exit, as depicted in Figure 10. The relationship between the road surface brightness of the threshold segment *L_th_* and the 20° field-of-view average external brightness *L*_20_ outside the tunnel is shown in Equation (3). In the formula, k denotes the proportional coefficient. The coefficient *k* quantifies the brightness compensation intensity required to overcome the black hole effect, and can further characterize the severity of the black hole effect indirectly.

*L_th_* = *k* ⋅ *L*_20_ (3)

**Figure 11:**
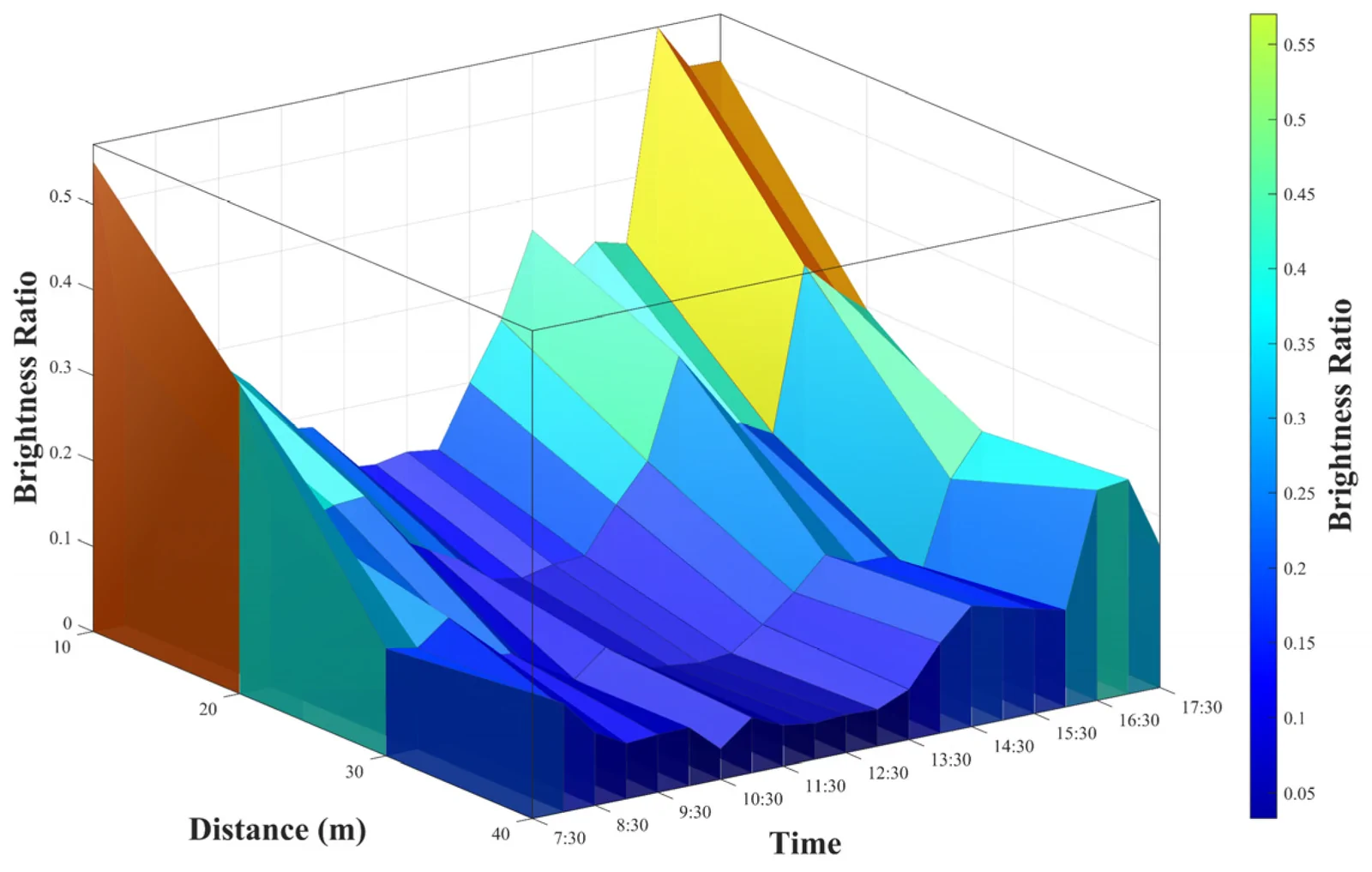
A three-dimensional dynamic evolution model of the intensity of the black hole effect in time–distance sequences.

Based on the above theoretical basis, this study develops a quantitative evaluation program for tunnel black hole effect intensity. For the collected time–distance sequential image dataset of tunnel entrances, four image frames are randomly selected for each sample category. An interactive manual bounding box selection method is adopted to delineate appropriate road surface regions inside and outside the tunnel for average brightness calculation. During the selection process, interference factors such as vehicles and road shadows are effectively eliminated to avoid their impacts on brightness statistics. Through batch statistical analysis, the evolution characteristics of tunnel black hole effect intensity varying with time and distance from the tunnel entrance are preliminarily explored, and a three-dimensional model is established as shown in Figure 11.

**Figure 12:**
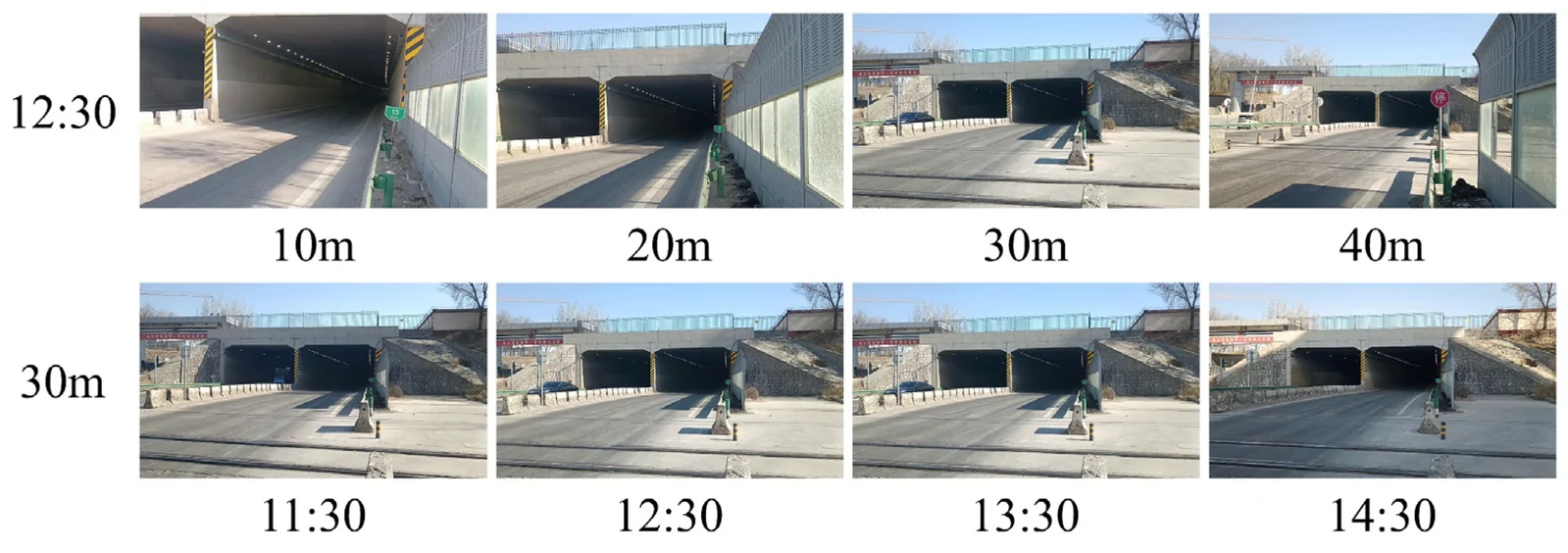
Display of the original statistical image depicting the minimum luminance ratio along with its corresponding adjacent time and distance.

In this three-dimensional dynamic evolution model, the *X*-axis represents the distance from the tunnel entrance, the *Y*-axis denotes time, and the *Z*-axis corresponds to the brightness ratio between the inside and outside of the tunnel. A smaller ratio indicates a more severe influence of the black hole effect. Within the observation period of 7:00–18:00, the natural light intensity outside the tunnel at 7:00 and 18:00 is lower than the supplementary lighting brightness inside the tunnel. The average brightness ratios at these two moments are both greater than 2, which fails to satisfy the generation conditions of the black hole effect. Thus, these two groups of data are excluded from the statistical sequence. During the valid observation period of 7:30–17:30, the internal and external brightness ratio of the tunnel presents an overall single-valley trend of decreasing first and then increasing, reaching the minimum value around noon, which is basically consistent with the conclusions of existing studies. An abnormal increase in the brightness ratio is observed at 14:30. Data attribution analysis reveals that this phenomenon is caused by the overall occlusion of the acquisition area by surrounding high-rise buildings, which significantly reduces the brightness difference between the inside and outside of the tunnel. From the spatial perspective, the influence of the tunnel black hole effect is not limited merely to the entrance area. As the distance from the tunnel entrance increases, the brightness ratio gradually decreases, and the influence degree of the black hole effect intensifies accordingly. In this observation, the minimum luminance ratio of 0.03 appears at 12:30 and 30 m away from the tunnel entrance, and the corresponding collected image is shown in Figure 12.

The above statistical analysis intuitively shows the time and distance intervals with strong black hole effect, which provides a theoretical basis for supplementing typical scenario data in subsequent data collection. Meanwhile, the three-dimensional dynamic evolution model constructed in this study overcomes the limitation of characterizing the tunnel black hole effect only by a single factor, and offers theoretical guidance for engineering practices including tunnel construction and energy-efficient operation of supplementary lights in tunnels.

## 3. Experiments

### 3.1. Experimental Environment and Parameter Settings

All experiments were conducted on a single-GPU cloud server. The hardware configuration consisted of one NVIDIA GeForce RTX 4090 GPU with 24 GB VRAM, a 16-core Intel Xeon Gold 6430 processor, and 120 GB system RAM. The software environment was built on the Ubuntu 22.04 LTS operating system, with the GPU computing stack established based on CUDA 11.8. PyTorch 2.1.2 was adopted as the deep learning framework, and Python 3.10 was used for programming. All model training and inference procedures were implemented under the aforementioned single-GPU hardware setup. Random seeds were fixed throughout all experiments to guarantee strict reproducibility of experimental results.

All input images were resized to 640 × 640 during preprocessing. The default training batch size was set to 16. Due to its large model size and VRAM constraints, the batch size of Yolo12x was adjusted to 8, which caused a slight impact on its training performance. The total training epoch was set to 300, and an early stopping strategy was enabled to mitigate overfitting. A cosine annealing learning rate scheduler was adopted for training under the transfer learning paradigm. For the training stage on the BH-Normal dataset, the initial learning rate was set to 0.001, and the final learning rate decayed to 1% of the initial value. A more conservative initial learning rate of 0.0001 was determined via preliminary experiments for the fine-tuning stage on the BH-Blackhole dataset. All experiments employed the SGD optimizer, for which the momentum and weight decay coefficients followed the model’s default settings (0.937 and 0.0005).

### 3.2. Performance Evaluation Metrics

This paper selects four indicators, namely Precision (P), Recall (R), mAP@0.5 and mAP@0.5:0.95, as the baseline evaluation standards for the dataset. The definitions and descriptions of each indicator are as follows:

(1) Precision (P)

Represents the proportion of real target samples among the samples predicted as targets by the model. The calculation formula is:

(4) $$\mathrm{Precision}=\frac{\mathrm{TP}}{\mathrm{TP}+\mathrm{FP}}$$

In Equation (4), TP (True Positive) is the number of positive samples correctly predicted by the model; FP (False Positive) is the number of negative samples misjudged as positive samples by the model.

(2) Recall (R)

Represents the proportion of all real target samples in the dataset that are successfully detected by the model. The calculation formula is:

(5) $$\mathrm{Recall}=\frac{\mathrm{TP}}{\mathrm{TP}+\mathrm{FN}}$$

In Equation (5), FN (False Negative) is the number of positive samples misjudged as negative samples by the model.

(3) mAP@0.5

The mean average precision of all detection categories when the Intersection over Union (IoU) threshold is set to 0.5.

(4) mAP@0.5:0.95

For all intervals of the IoU threshold ranging from 0.5 to 0.95 in 0.05 increments, calculate the average mAP for each threshold.

### 3.3. Experimental Results and Analysis

The baseline experiments based on the dataset constructed in this paper cover full-scale models of mainstream YOLO architectures. Three random seeds are configured for each experimental group to conduct repeated comparative trials, and all experimental results are reported in the form of mean ± standard deviation. All experimental data are authentic and fully reproducible.

Firstly, this paper adopts a transfer learning strategy to design the baseline Experiment N1. Based on the pre-trained weights provided by the official Ultralytics repository, all models are fine-tuned on the BH-Normal subset to accelerate model convergence and adapt to the object category recognition task of the constructed dataset. Three random seeds, i.e., 42, 123 and 3407, are adopted in this training process, and the trained models are aggregated into the BH-N model set. The test results of the BH-N model set on the BH-Normal subset demonstrate that the mAP@0.50 values of all YOLO models exceed 95%. In terms of mAP@0.5:0.95, the lightweight n-series models of each architecture remain around 80%, while all other models achieve values higher than 86%. This result verifies two key conclusions: first, BH possesses reliable annotation accuracy and high data quality; second, existing mainstream detection models exhibit superior object detection performance under traffic scenarios with normal illumination.

Secondly, test experiment N2 was designed to assess the BH-N model set on the BH-Blackhole subset. The experimental results reveal that the mAP@0.50 of all models drops by more than 10%. The tunnel entrance black hole effect imposes a more severe impact on mAP@0.5:0.95, with an average performance decline exceeding 20%. Experiment N2 adequately validates that models trained on conventional traffic datasets fail to meet the requirement of high-precision object detection in tunnel entrance black hole scenarios.

**Figure 13:**
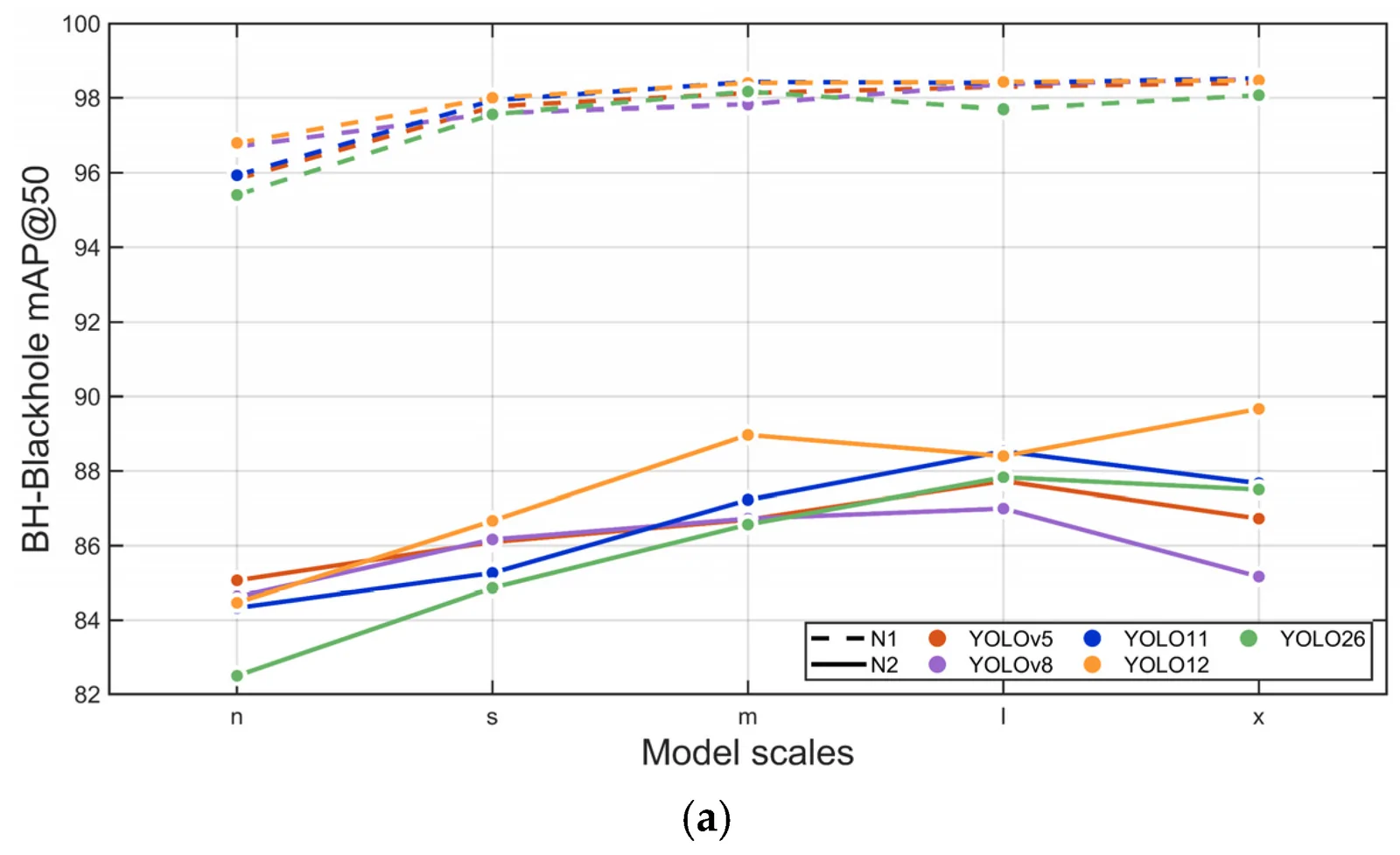

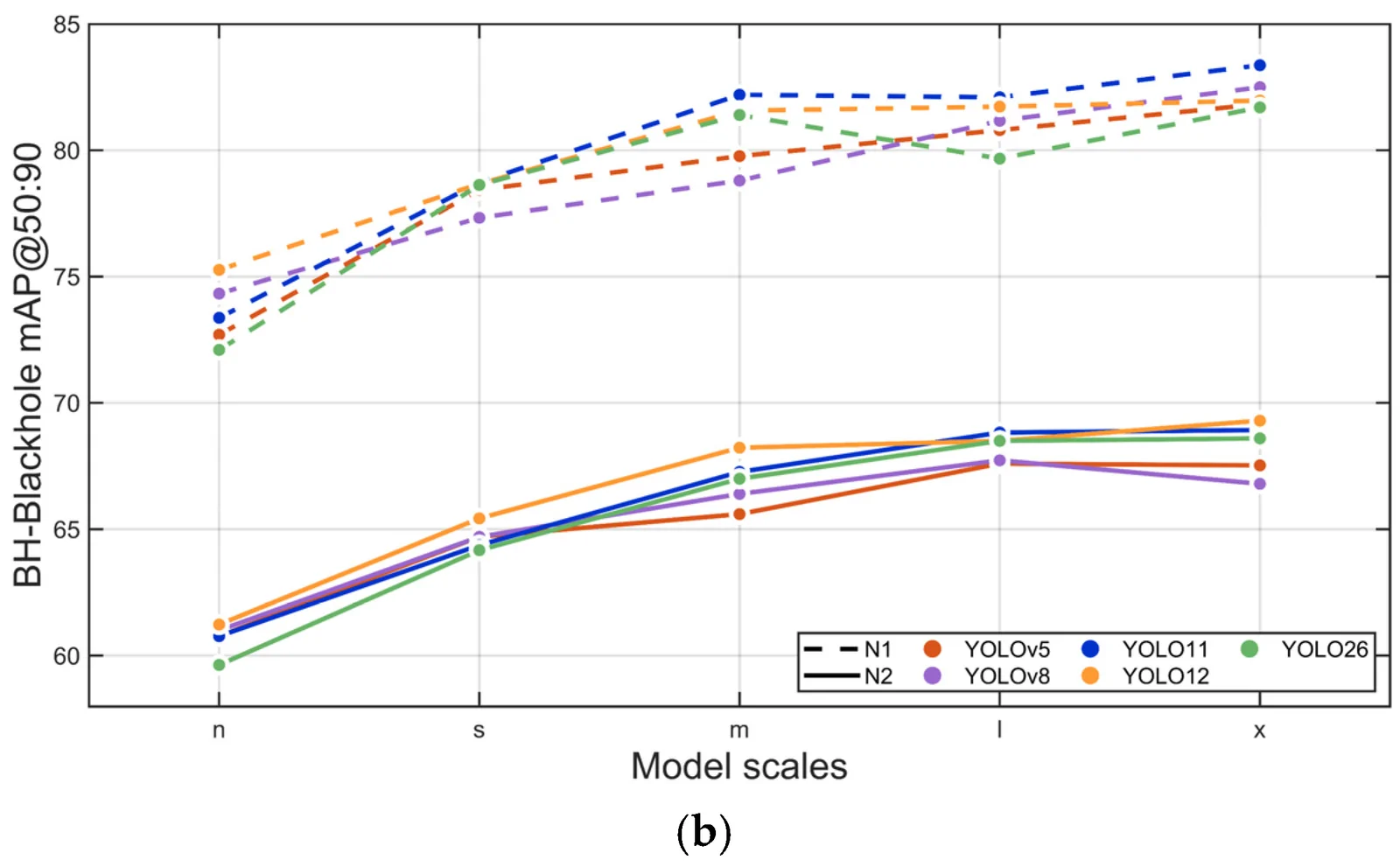
mAP experimental results for full-scale models of mainstream YOLO architectures. (**a**) Experimental results of mAP@0.5. (**b**) Experimental results of mAP0.5:0.95.

To alleviate the above performance degradation, Experiment B1 is designed in this work. The BH-N model set undergoes secondary enhanced training on the BH-Blackhole subset to obtain the BH-B model set. Random seeds of 1024, 2025, and 2026 are adopted for this training, and the trained BH-B models are subsequently evaluated on the BH-Blackhole test set. The precision and recall comparison between Experiment N2 and Experiment B1 is presented in Table 1. It can be observed that all evaluation metrics are effectively improved after the targeted enhanced training of the BH-N model set. The performance differences in mAP@0.5 and mAP@0.5:0.95 curves between the BH-N and BH-B model sets in Figure 13 intuitively reflect the performance variation before and after fine-tuning. As shown in Figure 13a, the mAP@0.50 of the BH-B model set rebounds substantially, with an average value above 95%. This indicates that YOLO models can achieve high-precision object localization in black hole scenarios by extracting fine-grained features, including structural characteristics of traffic objects (e.g., vehicle taillights) and color distinction between targets and backgrounds. As illustrated in Figure 13b, the mAP@0.5:0.95 of the BH-B model set rises to the range of 70–85% compared with the BH-N model set, achieving an obvious overall performance improvement. Among all models, YOLO11 achieves the optimal mAP@0.5:0.95 of 83.37%, which can preliminarily satisfy the object detection accuracy requirements of advanced driver assistance systems (ADAS).

**Figure 14:**
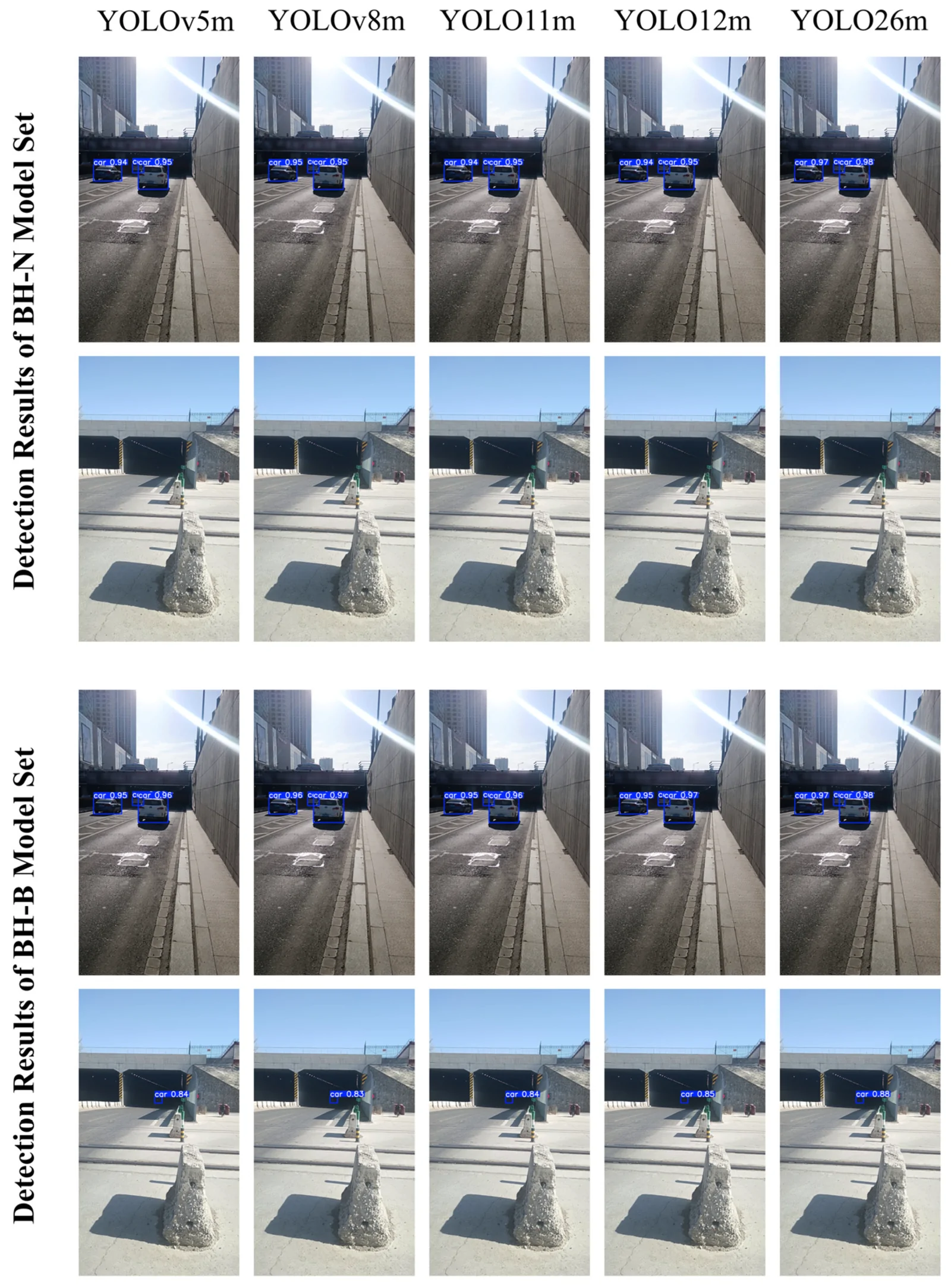
Test results for YOLO M-series models.

To intuitively demonstrate the performance improvement of YOLO models for object detection in the tunnel entrance black hole effect scenario brought by the BH, this paper selects the m-series models from mainstream YOLO architectures. These models can not only meet the real-time detection speed requirements of advanced driver assistance systems (ADAS) but also have high detection accuracy. All tested models are trained with a fixed random seed, and the optimal training weights are adopted for comparative analysis. As illustrated in Figure 14, models trained on the BH-N dataset before fine-tuning can accurately detect objects under normal illumination conditions. However, affected by the low-illumination interference caused by the tunnel entrance black hole effect, they completely fail to detect objects in the dark tunnel area. In contrast, the BH-B model set obtained by fine-tuning with the BH-Blackhole subset not only effectively improves the detection confidence of objects under normal illumination but also mitigates the low-illumination interference induced by the black hole effect, enabling effective detection of objects in dark tunnel regions.

### 3.4. Experimental Summary

As concluded from the above baseline experiments, the BH-Blackhole subset can effectively improve the object detection accuracy of YOLO models in the bridge and tunnel entrance scenarios with the black hole effect, which fully verifies the practical application value of this subset dataset. The baseline results of the dataset obtained in the experiments of this paper can serve as a reference benchmark for enhancing the object detection accuracy of YOLO models in black hole effect scenarios.

## 4. Conclusions

This study constructs a complete time–distance sequential image dataset for tunnel entrances. Through statistical analysis, a three-dimensional dynamic evolution model is established to characterize the variation in black hole effect intensity with time and distance, which preliminarily clarifies the evolutionary law of the black hole effect. The research provides theoretical support for dataset construction as well as engineering practices such as the layout of tunnel supplementary lighting lamps. In terms of engineering practice, this study improves the production workflow of YOLO datasets and provides a clear, complete and reliable standard procedure for subsequent researchers. Ultimately, this paper successfully establishes a dedicated YOLO dataset (BH) for bridge and tunnel entrance scenarios affected by the black hole effect, which effectively fills the research gap of existing datasets in this field. Meanwhile, E-BH adopts a management mode of in-depth correlation of features, partitioning and annotations, offering a demonstration paradigm for efficient dataset management. The self-developed TK in this research can also provide sustained tool support for the production, expansion and engineering application of subsequent datasets for bridge and tunnel scenarios. The baseline experiments in this paper adequately demonstrate that BH can substantially boost the object detection accuracy of full-scale mainstream YOLO models under black hole effect scenarios.

Due to limitations in data collection types and sample sizes, the established model for the evolution of tunnel black hole effect intensity cannot fully cover various actual working conditions. The current dataset is dominated by automobile-related targets, with a relatively small number of samples for other categories such as trucks and two-wheeler vehicles. There is a certain imbalance in the proportion of target categories, which to some extent affects the detection stability of YOLO for minority categories of targets. Future work will expand sample data under multiple constraint conditions, including different seasons (spring, summer and autumn), latitudes (low latitude and high latitude), and weather conditions (overcast, rainy and snowy days), with a focus on supplementing non-automobile target samples such as trucks and two-wheeled vehicles to alleviate the problem of imbalanced categories. Data filtering and frame segmentation may skew metrics like mAP towards optimism, but they do not affect the conclusions regarding changes in detection performance caused by the black hole effect or the accuracy of baseline metrics for the current dataset. Future work will introduce more representative challenging samples such as empty scenes, low-visibility frames, motion-blurred frames, and frames with extremely small targets at a distance, to test the robustness of the YOLO model under real-world conditions. Additionally, more images from unseen scenes will be collected to test the model’s generalization ability. It can be concluded from the baseline experimental results that although fine-tuning significantly improves the detection accuracy of existing YOLO models, there still remains considerable room for improvement in terms of mAP@0.5:0.95. Subsequent research will focus on low-illumination image enhancement and multi-scale feature fusion optimization, aiming to further enhance the object detection accuracy of YOLO algorithms in tunnel entrance and exit environments as well as various extreme illumination conditions. This research can provide technical support for the engineering implementation and performance iteration of visual perception schemes in autonomous driving systems.

## Figures and Tables

**Table 1:** Experimental Results on Precision and Recall for Full-Scale Models of Mainstream YOLO Architectures. (Note: N2 and B1 represent two test subsets of the BH dataset. P_{N2} and P_{B1} denote precision on subsets N2 and B1, respectively; R_{N2} and R_{B1} denote recall on subsets N2 and B1, respectively.)

Model	Scale	P_N2 (%)	P_B1 (%)	R_N2 (%)	R_B1 (%)
YOLOv5	n	86.83 ± 1.35	91.37 ± 0.38	75.7 ± 1.02	89.67 ± 0.85
	s	87.2 ± 0.14	93.47 ± 0.34	79.17 ± 0.59	92.5 ± 0.96
	m	87.77 ± 0.42	92.5 ± 0.22	78.83 ± 1.3	93.9 ± 0.75
	l	87 ± 0.73	93.5 ± 0.43	81.23 ± 0.42	93.77 ± 0.46
	x	87.47 ± 0.87	93.47 ± 0.47	81.53 ± 0.25	94.33 ± 0.21
YOLOv8	n	87.17 ± 0.54	92.23 ± 1.03	75.03 ± 0.48	91.07 ± 0.52
	s	87.83 ± 0.62	91.73 ± 0.66	77.7 ± 0.99	92.93 ± 0.56
	m	88 ± 1.1	92.23 ± 1.11	79.6 ± 1.8	93.37 ± 1.19
	l	86.63 ± 0.4	92.9 ± 0.29	81.7 ± 0.37	94.63 ± 0.05
	x	87.33 ± 0.69	93.5 ± 0.85	80.13 ± 0.6	94.9 ± 0.54
YOLO11	n	86.33 ± 0.78	92.47 ± 0.54	75.1 ± 0.96	89.47 ± 0.49
	s	86.4 ± 0.99	92.2 ± 0.36	78.43 ± 0.79	93.63 ± 0.38
	m	87.1 ± 0.96	93 ± 0.29	80.8 ± 0.14	94.37 ± 0.05
	l	86.17 ± 0.95	93.97 ± 0.49	83.37 ± 1.33	93.6 ± 0.37
	x	86.67 ± 0.71	94.03 ± 0.66	82.5 ± 0.54	94.63 ± 0.12
YOLO12	n	86.53 ± 0.37	92 ± 0.57	76.2 ± 0.45	91.4 ± 1.1
	s	88.23 ± 0.91	92.27 ± 0.62	79.4 ± 1.71	93.63 ± 0.41
	m	86.97 ± 1.3	93.67 ± 0.78	82 ± 0.75	94.27 ± 0.94
	l	87.47 ± 0.4	93.93 ± 0.54	81.93 ± 0.82	93.8 ± 0.22
	x	86.03 ± 1.11	93.53 ± 1.11	83.63 ± 1.31	94.37 ± 0.82
YOLO26	n	87 ± 0.51	89.6 ± 0.22	70.53 ± 0.87	87.43 ± 0.61
	s	86.3 ± 0.22	91.87 ± 1.16	76.37 ± 0.49	92.37 ± 0.99
	m	87.97 ± 0.59	91.87 ± 0.12	78.23 ± 0.73	94.3 ± 0.79
	l	86.8 ± 0.96	91.3 ± 1.31	80.23 ± 0.53	93 ± 0.41
	x	87.23 ± 0.31	92.07 ± 0.52	80.27 ± 0.21	93.77 ± 0.46

## Data Availability

The BH-N subset used in this study, including its complete training, validation, and test splits, is available for peer review via an anonymized link: https://pan.baidu.com/s/15XWESVVk3Q1Y0xX9yLQEag?pwd=7cdw (accessed on 6 October 2026). This link contains no author-identifying information and is compliant with double-blind review, and the subset is sufficient to fully reproduce the results of Experiment N1. The full BH dataset and the TK toolkit will be publicly released on GitHub(v1.0) under the CC BY-NC 4.0 license. Due to an ongoing patent application covering the dataset itself, the full dataset cannot be made publicly available at this time; we commit to releasing it within six months after the publication of the patent application, and no later than June 2028. Prior to public release, researchers may contact the corresponding author ([chenyun14@mails.jlu.edu.cn]) to request access for academic evaluation purposes.
